# OBIB-a novel ontology for biobanking

**DOI:** 10.1186/s13326-016-0068-y

**Published:** 2016-05-02

**Authors:** Mathias Brochhausen, Jie Zheng, David Birtwell, Heather Williams, Anna Maria Masci, Helena Judge Ellis, Christian J. Stoeckert

**Affiliations:** Department of Biomedical Informatics, University of Arkansas for Medical Sciences, 4301 W. Markham St., #782, Little Rock, AR 72205-7199 USA; Department of Genetics, Institute for Translational Medicine and Therapeutics, Institute for Biomedical Informatics, Perelman School of Medicine, University of Pennsylvania, Philadelphia, USA; Penn Medicine BioBank, Institute for Translational Medicine and Therapeutics, Perelman School of Medicine, University of Pennsylvania, Philadelphia, USA; Department of Biostatistics and Bioinformatics, Duke Medical Center, Duke University, Durnham, USA; Duke Biobank, Duke Translational Research Institute, Duke University, Durnham, USA

**Keywords:** Ontologies, Biobanking, Biorepository, Terminology

## Abstract

**Background:**

Biobanking necessitates extensive integration of data to allow data analysis and specimen sharing. Ontologies have been demonstrated to be a promising approach in fostering better semantic integration of biobank-related data. Hitherto no ontology provided the coverage needed to capture a broad spectrum of biobank user scenarios.

**Methods:**

Based in the principles laid out by the Open Biological and Biomedical Ontologies Foundry two biobanking ontologies have been developed. These two ontologies were merged using a modular approach consistent with the initial development principles. The merging was facilitated by the fact that both ontologies use the same Upper Ontology and re-use classes from a similar set of pre-existing ontologies.

**Results:**

Based on the two previous ontologies the Ontology for Biobanking (http://purl.obolibrary.org/obo/obib.owl) was created. Due to the fact that there was no overlap between the two source ontologies the coverage of the resulting ontology is significantly larger than of the two source ontologies. The ontology is successfully used in managing biobank information of the Penn Medicine BioBank.

**Conclusions:**

Sharing development principles and Upper Ontologies facilitates subsequent merging of ontologies to achieve a broader coverage.

## Background

The field of biobanking demands data integration. This need arises on multiple levels: institutional, cross-institutional, and sometimes even cross-national. Most institutions operate multiple biobanks that were established to fulfill diverse user requirements and therefore use different data representations and schemata. Integrating the data from these biobanks is a challenge at best and impossible at worst.

In recent years a number of projects have set out to answer the call for more specimen and data sharing across multiple biobanks and multiple institutions, both on a national and on a transnational level [1, 2]. One of the earliest examples of a transnational sample sharing project is the European Biobanking and BioMolecular Research Infrastructure (BBMRI) [3]. Using large amounts of data from multiple biobanks is an important way to enable statistical analysis, which can lead to uncovering associations between phenotypes and diseases [4]. The two key challenges are a) identifying specimens for research, and b) utilizing the existent wealth of information present in biobanks effectively by integrating data stored in those repositories [5, 6].

Andrade et al. have pointed out that semantically rich ontologies provide a promising approach to integrating data from diverse biobanks [7]. In 2013 Brochhausen et al. published Ontologized MIABIS (OMIABIS), a Web Ontology Language (OWL)-coded ontology for biobank administration based on use cases and competency questions derived from BBMRI (http://purl.obolibrary.org/obo/omiabis/merged/omiabis.owl) [8]. OMIABIS is one of the source ontologies that was used in creating the Ontology for Biobanking (OBIB). It is linked to the BBMRI effort [3] and is based on the Minimum Data Set for sharing biobank samples, information and data [9]. One of the key advantages of using ontologies, and in particular re-using pre-existing ontological representations is the possibility to link data from biobanks to other biological and biomedical repositories using common identifiers, e.g. from Gene Ontology [6]. This possibility will improve the utility of the shared biobank data tremendously since it allows easy retrieval of related data using semantic web technologies, such as RDF, SPARQL, and OWL. The capability to use biobank data in that way will make biobanks even more important for and accessible to translational research since the data already exist in a way that allows easy linkage to data across other disciplines from basic science to clinical research.

**Fig. 1:**
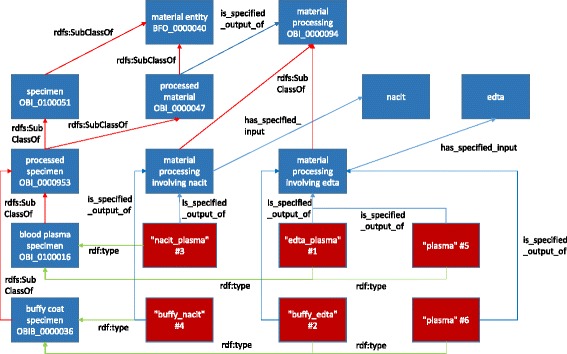
Representation of edta_plasma, buffy_edta, nacit_plasma, buffy_nacit, plasma, and buffy specimens according to the OBIB strategy. Blue boxes represent classes; red boxes represent individuals; red arrows represent rdfs:subClassOf; green arrows represent rdf:type; blue arrows represent OWL object properties (the labels are specified). While all OWL object properties link instance to instance, in this figure there are object properties connecting OWL classes to each other. This represents a property restriction on the source class with existential quantification (all-some restriction)

In addition, the use of a formal logical model to represent aspects of biobanking procedures and protocols prevents unnecessary complexity that can lead to cumbersome and error-prone data management and retrieval processes. One example of a complexity issue is a biobank protocol involving something as simple as specimen type. Consider a blood collection protocol that collects two vials of blood. Each vial contains an anti-coagulant, in this case one is ethylenediaminetetraacetic acid (EDTA) and the other monosodium citrate (NaCit). Each specimen goes through centrifugation producing a number of plasma and buffy coat specimens. The lab decides to tag their specimens in the following way: "edta_plasma", "buffy_edta", "nacit_plasma", "buffy_nacit". The "edta_plasma" and "buffy_edta" specimens are the derivatives of the EDTA parent specimen and analogously for "nacit_plasma" and "buffy_nacit" specimens. So the lab is encoding two pieces of information in the "specimen type", the parent specimen and the anticoagulant. Notice also that they chose the anticoagulant as the prefix for plasma specimens, but as the suffix for the buffy specimens, due to their perception that the anticoagulant is more important for the plasma specimens and less so for the buffy specimens. Another lab has a simpler protocol. They collect one EDTA specimen and produce plasma and buffy specimens. Since they collect only one type of blood specimen, they tag their specimens simply as "plasma" and "buffy". The information about the anticoagulant is implied as is information about the parent specimen. Imagine all these specimens end up in a unified biobank. The following specimen types would be present: edta_plasma, buffy_edta, nacit_plasma, buffy_nacit, plasma, and buffy. The inconsistencies are readily apparent. An ontology can provide a formal model for different attributes related to a specimen type, such as the parent specimen and the anticoagulant. Based on these attributes and others specimen types can be created as axiomatically defined classes (Fig. 1).

In this paper we present and describe the Ontology for Biobanking (OBIB). OBIB has been created by merging OMIABIS with a more specimen-focused biobank ontology developed at the University of Pennsylvania called the Biobank Ontology (BO). We provide an overview of the methodologies used to a) build both ontologies and b) merge them. We also give an outline of the domain covered by the newly created ontology and describe its current use. Finally, we discuss the next steps in expanding OBIB and linking it to ongoing efforts regarding biobank terminology. While we think that shared ontologies are one way to facilitate sharing information across multiple sites, the description and discussion of how to technically implement such a system (federated queries) are regarded as out of scope for this paper.

## Methods

OBIB is the result of merging two pre-existing ontologies, OMIABIS and BO. In this section we describe criteria and methodologies used by both the developers of OMIABIS and BO. We will give a brief overview over the two source ontologies and describe the merging process in detail.

Both ontologies, OMIABIS and BO are built based on the principles of the Open Biological and Biomedical Ontologies (OBO) Foundry (http://obofoundry.github.io/principles/fp-000-summary.html) [10]. Both are extensions of the Ontology of Biomedical Investigations (OBI) (http://purl.obofoundry.org/obo/obi.owl) [11]. OBI is based on Basic Formal Ontology (BFO), an Upper Ontology frequently used to represent biological and biomedical domains [12, 13]. One of the OBO Foundry principles is re-use of pre-existing ontologies or their classes and object properties in order to prevent multiple representations of the same entities. Both, OMIABIS and BO re-use numerous terms from existing ontologies. Both ontologies were developed based on a methodology that focuses on representation of the real-world phenomena that are described by the data, which is intended to be managed, instead of creating OWL representations of existing data schemata. Smith and Ceusters have coined the term ontological realism for this approach [14]. The example provided of specimen type shows that relying exclusively on pre-existing data representations can lead to problems integrating data from heterogeneous resources. Instead of providing representations base on the term used, such as "edta_plasma", "buffy_edta", "nacit_plasma", "buffy_nacit", "plasma", and "buffy", ontologies should represent the specimens, the parent specimens, the anticoagulants and the different processes that were necessary to create the specimen. Thus, individual specimens can be sorted into specimen types based on what their parent specimens are and which processes they passed through. This methodological paradigm fosters linking the biological sources to the specimens to the data about those specimens.

### OMIABIS

*Ontologized MIABIS* (OMIABIS) (http://purl.obolibrary.org/obo/omiabis/merged/omiabis.owl) was created as an OWL implementation of the BBMRI's Minimum Information About BIobank data Sharing (MIABIS). It is based on the BBMRI use cases, which are mostly population and cohort based. Due to juridical and ethical reasons searching individual specimens was out of scope for the initial implementation of OMIABIS [8].

The competency questions for the development of OMIABIS were:

- Which biobanks hold frozen specimens?
- Which biobanks hold blood, plasma and serum?
- Which blood plasma specimens are owned by one specific biobank organization?
- Which departments of a specific university have members that are serving as biobank contacts?
- What are the e-mail addresses of all biobank contact persons at one specific biobank organization?

These competency questions demonstrate that the focus of the OMIABIS development was less to retrieve information about individual specimens and to order those specimens, but more to obtain basic population-level and repository specific information about biobank administration and related study administration. OMIABIS represents both relevant objects, such as "biobank" or "biobank organization", and relevant processes, such as "specimen handling" and "sampling specimens for biobank". Terms were re-used from the Cell Type Ontology (CL), Chemical Entities of Biological Interest (ChEBI), Common Anatomy Reference Ontology (CARO), Information Artifact Ontology (IAO), NCBITaxonomy, Ontology of Medically Relevant Social Entities (OMRSE), Phenotypic Quality Ontology (PATO), Proper Name Ontology (PNO), and Reagent Ontology (REO). One hundred twenty-six entities were created specifically for OMIABIS resulting in a total of 428 classes, 15 individuals, 75 object properties, and 990 logical axioms. The ontology and additional details can be found at https://github.com/OMIABIS/omiabis-dev.

### Biobank ontology (BO)

Another biobank ontology based on OBI, BO, was generated independently of OMIABIS to address use cases provided by the Penn Medicine BioBank, which served along with OMIABIS as a starting point for OBIB. The competency questions for the development of BO were:

- How many study subjects have filled out a patient questionnaire for which there is an associated collection packet?
- What blood specimens are available from study participants? What chemical additive was used in the container?
- What is the storage state of the specimen of interest? How has the specimen been processed?

The BO aimed to address these competency questions by covering the processes along with inputs and outputs associated with a specimen in a biobank repository. These included ‘human subject enrollment’, ‘informed consent process’, ‘document editing’ (filling out case report forms), ‘specimen collection process’, ‘material processing’ of the specimen, ‘storage’ of the specimen, and shipping and handling processes. Patient-related terms (e.g., ‘smoking behavior’) were also included. Terms were re-used from BFO, IAO, OBI, ChEBI, EFO, NCBITaxon, OGMS, PATO, and UBERON. Approximately 50 terms were added to address biobank specific needs (e.g., collection packets for specimens) resulting in 227 classes, 18 individuals, 34 object properties, and 526 logical axioms. The ontology and additional details can be found at https://github.com/biobanking/Penn-Biobank.

### Methodology of merging OMIABIS and BO

The fact that both OMIABIS and BO were built based on OBI and used BFO as top-level ontology greatly facilitated the integration of the two ontologies. However, various BFO versions were used and some common terms were included in both OMIABIS and BO. The following processes were performed before integration of OMIABIS and BO:

1. Converted both ontologies using BFO version 2.0 Graz release (http://purl.obolibrary.org/obo/bfo/2014-05-03/classes-only.owl). The conversion was made using BFO converter (http://bfoconvert.hegroup.org/).
2. Separated terms defined in OMIABIS or BO from those defined in external resources (OBO Foundry Ontologies) and saved those in different OWL files.
3. Identified terms defined in both OMIABIS and BO and merged the overlapping terms where necessary.

This pre-processing of OMIABIS and BO before merging resulted in the following files:

- omiabis.owl or biobank.owl: OMIABIS or BO specific terms.
- import_OBI_subset.owl: OBI subset upon which OMIABIS or BO was built containing terms needed from both IAO and OBI. The OBI subset was retrieved using Ontodog, a tool that can retrieve a set of terms of interest and all related axioms from a source ontology [15].
- import_OBO.owl: terms defined in external OBO Foundry ontologies and retrieved using OntoFox, a tool that can retrieve terms of interest from a source ontology based on MIREOT mechanism [16].
- externalByhand.owl: terms defined in external OBO Foundry ontologies added manually.

These files are available on the OMIABIS project website:

OMIABIS: https://github.com/OMIABIS/omiabis-dev/tree/master/BFO2%20omiabis and BO: https://github.com/OMIABIS/omiabis-dev/tree/master/biobank-omiabis/BFO2%20biobank.

Since OMIABIS and BO focused on different aspects of biobanking, no overlap in terms was found between OMIABIS and BO specific terms (omiabis.owl and biobank.owl).

In preparing BO for merging it with OMIABIS, we also compared BO to other OBO Foundry ontologies relevant to the domain. We found a few BO terms related to informed consent that overlapped with terms from the Informed Consent Ontology (ICO) [17]. These BO terms were replaced by ICO terms. Since BO was not officially registered as an OBO Foundry community, BO specific terms were assigned OBIB term identifiers after merging.

Protégé was used to perform the merging of the OWL files prepared based on the two ontologies. The process was a series of merges for pairs of equivalent OWL files used by OMIABIS and BO to create: import_OBI_subset.owl from subset OWL files, import_OBO.owl from OWL files of external terms retrieved using OntoFox [16], externalByhand.owl from manually imported external terms OWL files, and biobank-omiabis.owl from omiabis.owl and biobank.owl. The one remaining BFO 1.1 class, ConnectedTemporalRegion, was dealt with by taking advantage of its definition as equivalent to the union of its two subclasses, temporal_instant and temporal_interval. These have both been mapped to BFO 2.0 classes, zero-dimensional temporal region and one-dimensional temporal region, which were used to replace ConnectedTemporalRegion.

These merged OWL files are available on github: https://github.com/OMIABIS/omiabis-dev/tree/master/biobank-omiabis.

The merged OMIABIS and BO ontology is available on:

https://raw.githubusercontent.com/OMIABIS/omiabis-dev/master/biobank-omiabis/biobank-omiabis_merged.owl.

Finally, the consistency of the merged ontology was checked using Hermit 1.3.4 and no inconsistencies were found.

## Results

### Ontology of biobanking (OBIB)

The first release of OBIB (http://purl.obolibrary.org/obo/2014-09-22/obib.owl) was made based on the OWL files described in section Methodology of merging OMIABIS and BO. The development of OBIB followed the OBO Foundry principles. OBIB is freely and openly available. The latest release can be obtained from http://purl.obolibrary.org/obo/obib.owl. The community driven development is done using an open code repository, https://github.com/biobanking/biobanking. Issues and term requests can be communicated at https://github.com/biobanking/biobanking/issues. Currently, OBIB contains 516 classes including 126 OMIABIS and 46 OBIB classes, 19 individuals, 83 object properties, and 1172 logical axioms.

One of the central terms of OBIB is *biobank*. This term was merged from OMIABIS. OBIB defines biobank as:

"A biobank is a collections of samples of biological substances (e.g. tissue, blood, DNA) which are linked to data about the samples and their donors. They have a dual nature as collections of samples and data".

The equivalent class axiom, which provides a formal machine-parsable definition, can be accessed at: http://www.ontobee.org/ontology/OBIB?iri=http://purl.obolibrary.org/obo/OMIABIS_0000000.

From these definitions it is obvious that from the perspective of OBIB, a biobank consists of both the specimens and the data about the specimens and the specimen collections. OBIB differentiates between a biobank and a biobank organization. The latter is defined as:

"An organization bearing legal personality that owns or administrates at least one biobank."

This differentiation enables concise representation of an organization running more than one biobank, as is regularly the case for hospitals, research facilities and others. So, OBIB fills gaps regarding the representation of biobanking that have so far existed in OBO Foundry ontologies and other ontologies.

Due to the fact that OBIB is the result of merging two independently developed ontologies, coverage is broad. Naturally, it covers information about specimens and processes like specimen collection, specimen handling and storage. It also represents donors and patients and medical record data pertaining to those. In addition, it provides representation for clinical studies and specimens and data accrued during those. In the current release, the OBIB-generated terms are related to the containment of the specimen (e.g., OBIB_0000028: collection packet), additives (e.g. OBIB_0000022: blood additive role), and storage mechanisms (e.g., OBIB_0000030: blood spot card). OBIB specific terms were also generated for patient forms (e.g., OBIB_0000017: data confirm questionnaire) and associated health-related questions (e.g., OBIB_0000053: duration time of smoking).

**Fig. 2:**
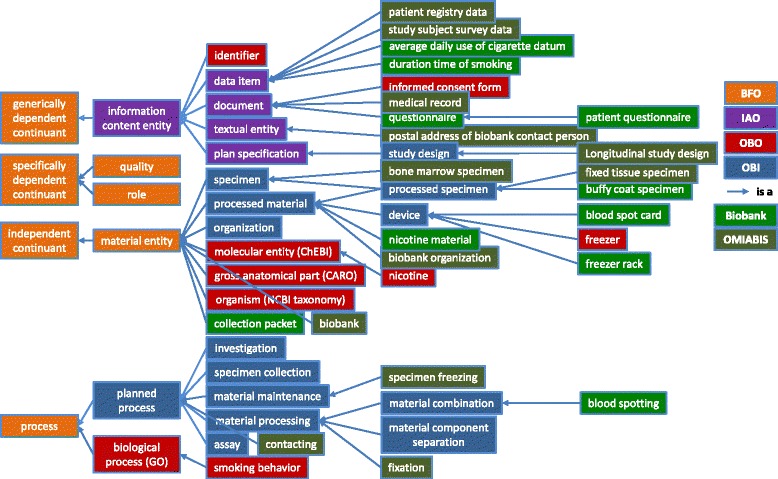
Selection of central classes of OBIB and their superclasses. The leftmost four BFO classes are subclasses of further BFO classes which are not shown here for readability

Most terms needed by OBIB are not specific to biobanks and available from other OBO ontologies and imported. Figure 2 shows a selection of the most relevant classes of OBIB and their superclasses. The figure shows that many classes in OBIB are re-used from other commonly used ontologies of the biomedical domain, such as OBI, IAO, and others, or subclasses of those classes. Notably, some of those are central classes such as "specimen" and "specimen collection", both re-used from OBI. Other OBI classes, such as "organization" and "material maintenance" are superclasses for classes highly relevant to the biobanking domain, such as "biobank organization" and "specimen freezing".

This not only highlights the relevance of OBI for the representations that are part of OBIB, but also provides an important opportunity regarding the use of semantic web technologies in translational research. Terms like "specimen" are used by multiple ontologies. The National Center for Biomedical Ontologies' (NCBO) BioPortal [18], an ontology lookup service, retrieved 18 different representations of the term "specimen" in BioPortal ontologies (when queried Nov. 18, 2015). OBI's representation of specimen is one of them. However, the only specimen representations that are referred to by other ontologies are the OBI representation (referenced by 11 other ontologies) and the Semanticscience Integrated Ontology (SIO) [19] representation (referenced by 1 other ontology). This shows that the representation of specimen by OBI is by far the one most widely used by other ontologies. Re-use of OWL entities (such as "specimen") in multiple ontologies and in multiple applications using those ontologies is relevant, since the representation comes with a Unique Resource Identifier (URI) that allows linking data in RDF. This is a key strategy of semantic web technology that holds huge promise for the translational science community. Data created for use in a specific domain (e.g. biobanking) can be linked easily with data created in another domain (e.g. digital pathology) by using the same URIs to refer to the same entities.

### Usage of OBIB

At the Penn Medicine Biobank, OBIB is used as the semantic framework for a search system that supports cohort identification and deep data mining of the information associated with biobank specimens and specimen donors. We explored how OBIB could be used through a specific competency question of case/control matching. For cases, we wanted to identify patients who were consented to the biobank, had a history of type 2 diabetes, took a particular statin medication on or before the date of recruitment, and had a banked EDTA blood specimen. Eligible controls needed to have type 2 diabetes, have no history of taking any statin medications, and have a banked EDTA specimen. Controls needed to be matched to cases by gender, age at the time of recruitment, and Body-Mass Index (BMI). The end goal was an integrated graph database that contained the instances and semantics of our data and could be queried to answer our competency question, one which exemplified a typical request made about biobank data.

The data needed to answer the competency question were located in several relational data sources. Diagnosis and prescription data were stored in relational data sources derived from the patients' electronic medical record. Dates of birth and recruitment, gender, and BMI at time of recruitment were gathered in case report forms (CRF) at the time of enrollment. There were several iterations of the CRFs that were stored in databases with different table structures. A snapshot of the inventory data was generated from the specimen inventory system.

**Fig. 3:**
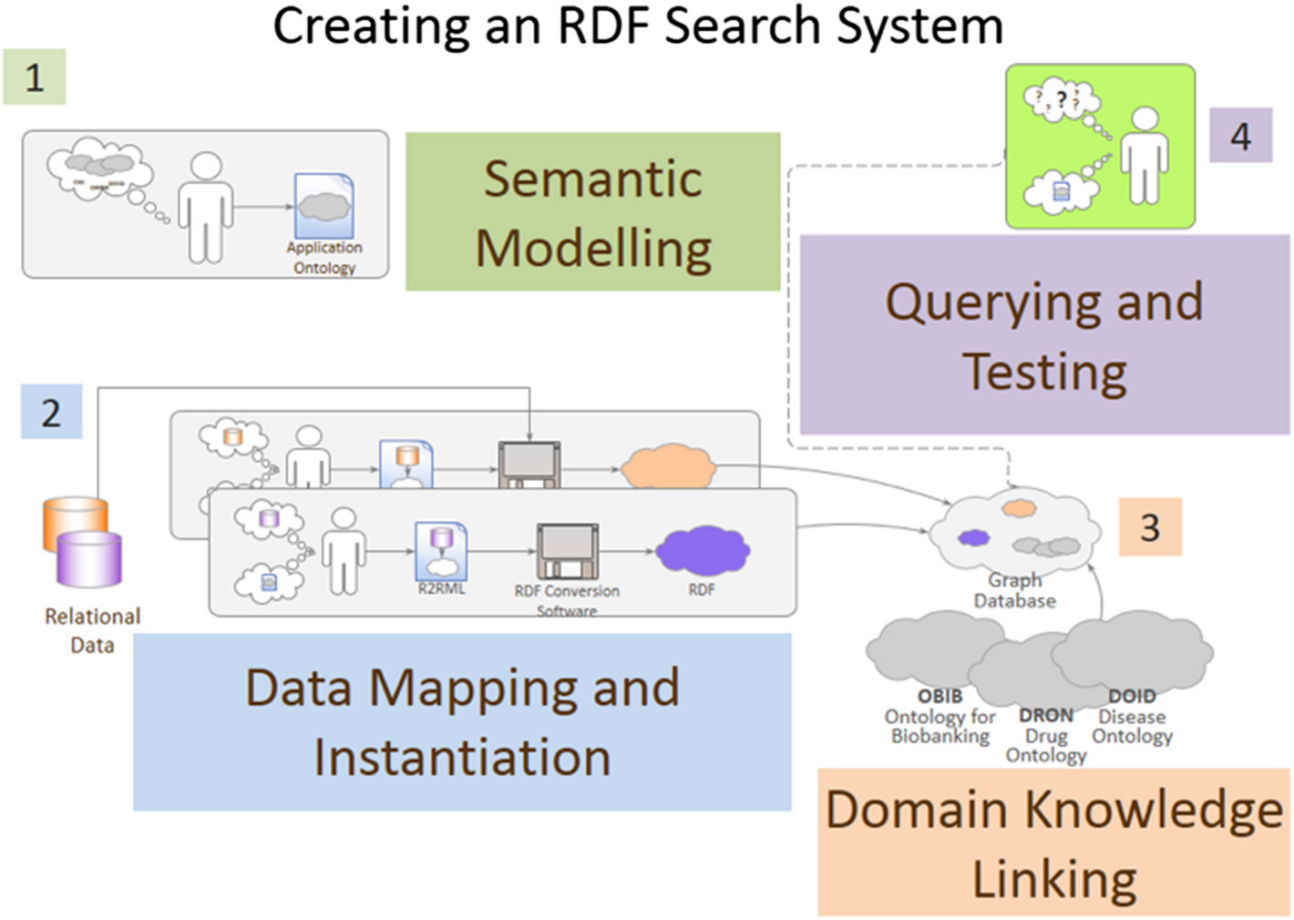
The process used for building the prototype RDF search system to answer the Penn Medicine Biobank case/control competency question. 1. Semantic Modeling-Ontology models are developed to model the semantics of the relational data and any OBO ontologies that are relevant to the data sources and potential queries. 2. Data Mapping and Instantiation-The models developed in step 1 are used to write mapping files to concretely map the relational data as RDF. Software tools to use these maps to instantiate the relational data as RDF data. 3. Domain Knowledge Linking-The instantiated RDF data and any relevant OBO Foundry Ontologies are loaded into a graph database. 4. Querying and Testing-Queries over the graph data can be created by referencing the OBIB model. To test, equivalent queries against the graph data and relational data are constructed and run to ensure data correctness

The process of building an RDF search system to answer our question was divided into 4 parts: 1) semantic modeling, 2) data mapping and instantiation, 3) domain knowledge linking, and 4) querying and testing (Fig. 3).

For semantic modeling, local domain experts and OBO Foundry ontology experts generated an ontology model using OBIB that included the portions of OBO Foundry ontologies relevant to the data sources. In our case this took the form of several Cmap [20] documents. Each data source was mapped separately and encapsulated the relational data, the semantics intrinsic in the relational data and any relevant domain knowledge. An additional model was created to show how the separate data sources were related. This model served as a guide to adding additional data sources and to the naming convention of International Resource Identifiers shared between data sources.

During data mapping and instantiation, the ontology models were referenced to generate a concrete map of how the relational data would be transformed into triples. This mapping can be expressed in RDB to RDF Mapping Language (R2RML) (http://www.w3.org/TR/r2rml), a language developed by the World Wide Web Consortium. There are several software conversion tools that use R2RML to instantiate relational data as RDF triples. In our case, we used D2RQ [21] as the conversion software and a D2RQ specific mapping language that is a derivative of R2RML.The ontology models were updated as necessary while writing and testing the conversion files.

Domain knowledge linking involved loading the instantiated RDF data and any related OBO ontologies into a graph database. We used Stardog (http://www.stardog.com) as our graph database into which we loaded RDF instance data and OBIB.

Querying and testing involved verifying the instantiated data were correct and answering the competency question. Equivalent queries were generated against the relational and graph data to ensure the data were accurately modeled.

## Future work

Recent research has shown that in spite of aiming to foster clear and concise class representation, definitions from ontologies do not always rate well with domain experts [22]. This highlights the need for closer collaboration between ontology curators and domain terminology experts regarding definitions, term descriptions and real life applicability. Therefore, the OBIB developers have started a collaboration with domain experts heavily engaged in the area of biobank terminology, the Duke Biobank, a consortium organization within the Duke Translational Research Institute. The Duke Biobank led the effort to select and implement a commercial biospecimen information management system (BIMS) to integrate information from Duke’s diverse biobanking entities. A priority at the outset of the selection process was the establishment of the policy that all biobanks participating in the BIMS must use a common terminology. To that end, terms were identified and defined through a consensus driven process with biobanking domain experts across the Duke campus. Sources considered for terms included publications related to biobanking terminology as well as related to biobanking pre-analytical variables, existing data elements in use in the existing biobanks, a public comment period, and out-of-the-box terms from the commercial BIMS, once it was purchased. The 18 month effort overall resulted in over 500 data elements covering the lifecycle of the biospecimen, as defined by the National Cancer Institute [23]. In an effort to further expand and share Duke’s work, a collaboration between Duke and OBIB began in the fall of 2014. Currently the collaboration is focused on the comparison of terms and classes between the Duke terminology and OBIB in an effort to identify intersections and gaps while implementing OBIB classes, with the goal of extending OBIB to cover the use cases underlying the development at Duke. To date, this approach has resulted in two different outcomes:

i) A Duke term mapped exactly the OBIB term but the naming was different (e.g. "collect" in Duke terminology correspond to "specimen collection" in OBIB).
ii) Duke term was not present in OBIB (e.g. Duke term "sample set" and "sample family").

This led to the creation of new terms specifying already existing classes in OBIB and highlighted the demand for additional general classes in OBIB (e.g., OBI_0002080: human specimen set; OBI_0002077: specimens derived from shared ancestor). The aim of this close interaction between the additional ontology users (Duke) and OBIB is to create a resource fulfilling the requirements of heterogeneous users. The value of this collaboration is in joining a robust biobanking terminology developed for a specific institution’s use, with a biobanking ontology created at two other institutions, leveraging domain knowledge in both biobanking and ontology in order to establish a single, relevant ontology resource with broad coverage to be applied to the field of biobanking.

Another key aspect of biobanking is related to informed consent and retrieving information about the consent given by the donors to facilitate research. We have already pointed out that OBIB already contained a basic representation of informed consent from the ICO. However, in order to retrieve the actual content of the consent it is not sufficient to limit the representation to the consent documents and the consent process. It will also be necessary to represent the rights and obligations that are created through the consenting. We have begun working with the developers of ICO to address this issue in the context of biobanking. While the development of an in depth representation of informed consent for biobanking is still ongoing, one strategy that has been identified as key, is using a pre-existing ontology that allows the representation of rights and obligations and the socio-legal processes that give rise to them. The fundamental aspects of this have already been addressed by the Ontology of Document Acts (d-acts) (http://purl.obofoundry.org/obo/iao/d-acts.owl) [24, 25].

## Conclusion

We have seen that for domains as central as biobanking more than one ontology might exist. In order to foster semantic integration of data for the largest possible number of users and consumers, it might be necessary to merge two or more ontologies.

In this paper we demonstrated that merging ontologies that share a common design methodology, and that extend the same Upper Ontology and Reference Ontology can be done fairly easily and with consistency. Both OMIABIS and BO adhere to OBO Foundry principles and were created based on the methodological paradigm of ontological realism. Both ontologies are extensions of OBI, which is based on BFO.

We have also demonstrated how the result of the merger is currently used and allows answering competency questions based on real-world use cases in the Penn Medicine Biobank. Finally, we illustrated how OBIB can serve as a means to capture the semantics and share the value of terminologies developed for institutional biobanks.

## Abbreviations

BBMRI: Biobanking and BioMolecular Research Infrastructure
BFO: basic formal ontology
BIMS: biospecimen information management system
BMI: body-mass index
BO: biobank ontology
CARO: common anatomy reference ontology
ChEBI: chemical entities of biological interest
CL: cell type ontology
CRF: case report form
EDTA: ethylenediaminetetraacetic acid
IAO: information artefact ontology
ICO: informed consent ontology
IRI: International Resource Identifier
MIABIS: minimum information about biobank data sharing
MIREOT: minimum information to reference an external ontology term
NaCit: monosodium citrate
NCBO: National Center for Biomedical Ontologies
OBI: Ontology for Biomedical Investigations
OBIB: ontology for biobanking
OBO: open biological and biomedical ontologies
OMIABIS: Ontologized MIABIS
OMRSE: ontology of medically relevant social entities
OWL: web ontology language
PATO: phenotypic quality ontology
PNO: proper name ontology
RDF: resource description framework
REO: reagent ontology
SIO: semanticscience integrated ontology
SPARQL: SPARQL protocol and RDF query language
URI: unique resource identifier
